# Enhancing Transitions of Care: A Cross-Sectional Observational Study on the Role of Clinical Pharmacists in Transition Management in a Latin American Hospital

**DOI:** 10.7759/cureus.68998

**Published:** 2024-09-09

**Authors:** Esteban Zavaleta-Monestel, Zoe Rojas-Barrantes, José Pablo Díaz-Madriz, Sebastián Arguedas-Chacón, Eugenia Cordero-García, José Chaverri-Fernández

**Affiliations:** 1 Pharmacy, Hospital Clinica Biblica, San Jose, CRI; 2 Pharmacy, Hospital Clínica Bíblica, San José, CRI; 3 Pharmacology, Toxicology, and Drug Dependence, Universidad de Costa Rica, San José, CRI

**Keywords:** clinical pharmacist, discharge counseling, medication reconciliation, patient outcomes, transition of care

## Abstract

Background

The seamless management of transitions of care (TOC) is necessary for patient safety, as it directly correlates with a heightened risk of medication errors and adverse effects. Clinical pharmacists emerge as key stakeholders in optimizing medication management during TOC, specifically during hospital admission and discharge, through the implementation of innovative programs that contribute significantly to the mitigation of medication errors and improve patient satisfaction.

Aim

This study aims to assess the benefits of pharmacist-led interventions in a Costa Rican private hospital's TOC program for polymedicated and high-risk patients during admission and discharge by identifying and addressing medication errors.

Methods

A cross-sectional observational study was conducted at Clínica Biblica Hospital in San José, Costa Rica, from February 2022 to May 2023 and focused on polymedicated patients with chronic therapy and high-risk medications. The TOC Medication Program was specifically implemented to focus on medication reconciliation during the admission and discharge processes. A clinical pharmacist documented interventions based on discrepancies found within each patient’s medication and assessed the economic impact of interventions on healthcare personnel during discharge by projecting potential complications in the absence of such interventions, a process that was validated by an internist physician.

Results

During the medication reconciliation at admission, medication discrepancies, mostly intentional omissions, were successfully addressed by clinical pharmacist interventions with a 90.2% acceptance rate during the admission process. At discharge, 18.9% of medications were high-risk, and nearly 40% of discharges were linked to drug-related problems (DRPs), prompting pharmaceutical interventions. The economic analysis indicated potential savings of $21,010.20 during discharge, demonstrating the substantial impact of interventions in preventing emergency service visits, specialist consults, and hospital admissions.

Conclusion

Pharmacist-led TOC programs offer important clinical advantages by effectively preventing and rectifying medication discrepancies. These discrepancies, if left unaddressed, pose a potential threat to patient safety. Moreover, the implementation of such programs demonstrates promising economic benefits.

## Introduction

According to the World Health Organization, transitions of care (TOC) are defined as "the various points where a patient moves to, or returns from, a particular physical location or makes contact with a health care professional to receive health care" [1]. Each transition point in a patient's care represents a high-risk scenario concerning patient safety, as documented evidence indicates increased medication errors and adverse effects [2].

Medication discrepancies during care transitions have been observed across diverse healthcare settings, including hospitals, community care, and long-term care facilities, occurring at different stages of care [3,4]. Research has found that around 30% of patients discharged from the hospital experience at least one medication inconsistency, and inadequate medication reconciliation processes during care transitions contribute to over 40% of reported medication errors. Additionally, up to 20% of patients encounter adverse events within three weeks of discharge, with a significant 60% of these being associated with medications and deemed preventable [3,5]. This underscores the critical need for implementing effective strategies to mitigate medication-related challenges throughout care transitions.

The shortcomings in care transitions not only compromise patient safety but also exert a strain on health systems. A significant consequence of inadequate transitional care is the high incidence of hospital readmissions. According to Medicare and Medicaid Services data, 2.6 million seniors are readmitted within 30 days after discharge, incurring an annual cost exceeding $26 billion [6]. Moreover, projections until 2020 estimated that around 27% of hospital readmissions in the United States could have been potentially avoided, resulting in an economic burden ranging from $25 billion to $45 billion annually [7].

Optimizing medication management is a critical aspect of healthcare that focuses on maximizing therapeutic outcomes while minimizing the risk of adverse events. In the complex TOC landscape, healthcare professionals must collaborate effectively to ensure the safety and well-being of patients. One key player in this process is the clinical pharmacist, whose interventions can significantly enhance the management of medications during TOC [1,8,9].

In recent years, innovative pharmacist-initiated transitional care medication programs have emerged to address these challenges in traditional care models, leveraging pharmacists' expertise in medication therapy management for targeted interventions. By examining the evidence supporting these programs and exploring strategies for implementation, healthcare systems can optimize medication management and enhance patient outcomes, especially in high-risk patients [1,8,10-15]. Clinical pharmacists are essential for proper medication use, addressing drug interactions and side effects. Collaborating with healthcare professionals, they ensure comprehensive medication management and personalized care plans, enhancing patient safety and reducing issues during transitions of care. They also conduct pharmacotherapeutic reviews to assess for duplications, interactions, and incorrect dosages. [1,7,8,10,11]

These initiatives have significant implications, reducing readmission rates and mitigating medication errors. For example, a study implementing a pharmacy heart failure resident-run TOC service for heart failure patients demonstrated a notable decrease in readmission rates by focusing on optimizing medication therapy and providing patient education [16]. Moreover, Ni et al. implemented a study that showed that a post-discharge pharmacy-led TOC program can reduce readmission rates at 30 and 180 days [17].

Yahya et al. conducted a systematic review, revealing that medication review and reconciliation stand out as the most frequently employed interventions by clinical pharmacists, particularly in post-discharge scenarios. Additionally, the authors also highlight the need for further exploration into the evolving role of clinical pharmacists within multidisciplinary teams. While their involvement continues to broaden, it remains crucial to delve deeper into the impact of pharmacist engagement across different care transitions, including both clinical outcomes and economic considerations [18].

This necessity is particularly pronounced in regions like Latin America, where research on care transition concepts is underdeveloped [19]. There is a notable lack of evidence regarding the functions of pharmacists in these processes and their potential benefits within these regions.

This study aims to assess the benefits of pharmacist-led interventions in a Costa Rican private hospital's TOC program for polymedicated and high-risk patients during admission and discharge by identifying and addressing medication errors.

This article was previously posted to the Research Square preprint server on July 2, 2024.

## Materials and methods

Study design

A cross-sectional observational study was conducted at Clínica Biblica Hospital, San José, Costa Rica, from February 2022 to May 2023. Patients at admission were eligible if they met the following criteria: polymedicated status (with a prescription for five or more medications [20]), undertaking chronic therapy before hospital admission, and use of high-risk medications during the study period. The classification of medications as high-risk or not was based on the list compiled by the Institute for Safe Medication Practices (ISMP) [21]. 

The medication review processes occurred in two distinct temporal stages; admission was conducted first, followed by discharge, utilizing the same methodology but with different sets of patients, meaning that patients were not monitored during both stages simultaneously.

Data were primarily extracted from electronic clinical records, which encompassed demographic details such as sex and age, the primary reason for hospital admission, chronic health conditions, results of laboratory tests, and medication records. This included active medications administered during the hospital stay, with detailed information on indication, dosage, frequency, and route of administration, as well as medications prescribed for chronic conditions, similarly outlined with their respective details. Furthermore, supplementary insights were gained through verbal interviews with patients and their associated support networks.

Medication reconciliation and analysis upon admission

The reconciliation process commenced with an interview conducted with the patient or their healthcare provider upon admission, as detailed in Appendix A. During this interview, inquiries were made regarding chronic pathologies and medications the patient regularly uses. This was undertaken to validate and enhance the patient’s electronic medical record, which was subsequently completed with information collected and recorded by other interdisciplinary team members, including physicians and nurses. The interviews adhered to the following criteria: Patient interviews were conducted within 24 hours of hospital admission and conducted individually with the patient. Caregivers may be present during interviews when necessary. If the patient cannot be interviewed due to their condition, interviews were conducted solely with caregivers; interviews were exclusively conducted by a qualified clinical pharmacist.

Information collected during the interview and the complete list of regularly used medications were documented on paper forms for subsequent entry into the patient’s electronic medical record. The parameters and information of interest included the patient’s medical history (comorbidities, allergies, previous hospitalizations) and, for each chronic medication, the following details were noted: active ingredient, dose, frequency, route of administration, and other specific indications according to each patient’s circumstances. A comprehensive list of medications was formulated based on the aggregated data. This information was compiled to provide better clinical follow-up to patients during their hospital stay. Additionally, a thorough understanding of the patients allows for more personalized follow-up and can even identify areas for improvement in the patient’s chronic treatment.

Medication reconciliation and analysis upon discharge

Upon the completion of the patient’s hospital stay and the issuance of the discharge prescription by the physician, patients became eligible for the discharge medication analysis. This analysis involved a thorough examination of the prescribed medication to identify any discrepancies or opportunities to improve pharmacotherapy.

Detected discrepancies were noted and categorized as drug-related problems (DRPs), further subdivided into categories of indication, effectiveness, safety, and adherence. Prioritization of interventions was conducted based on DRP categories, recognizing that this process varies according to the unique needs and circumstances of each patient. The clinical pharmacist delivered interventions to healthcare staff and patients to address the identified DRPs. The outcomes of these interventions, whether accepted or rejected, were documented. Results were stratified based on guidelines established by the Pharmaceutical Quality Alliance (PQA) Measurement Development Team (MDT) [22]. Additionally, the potential severity of the problem was determined on a scale of one to five, representing the lowest to highest clinical consequences to the patient related to DRPs, as per the guidelines of the National Coordinating Council for Medication Error Reporting and Prevention (NCC MERP) [23].

During this process, data collection was performed using a custom-designed tool by the clinical pharmacist overseeing the medication review process. This tool was specifically designed to collect pertinent information needed to conduct a comprehensive assessment of medication-related factors during care transitions. The tool covers information such as prescribed medications, patient demographics, medical history, and any medication-related issues identified. Additionally, it includes sections to document interventions made by the clinical pharmacist to address identified discrepancies or optimize pharmacotherapy (Appendix A).

Economic implications of interventions

Following the implementation of interventions with healthcare professionals during hospital discharge, a comprehensive analysis was conducted to identify potential complications that might have arisen in the absence of these interventions.

This analysis involved a meticulous review of patient records and discharge plans, focusing on identifying instances where the interventions could have prevented adverse outcomes such as medication errors or patient non-compliance, which could have led to emergency care visits, hospital readmissions, and additional specialist outpatient consultations.

To ensure the clinical relevance of these scenarios, they were subsequently reviewed and validated by an internist physician with extensive experience in hospital medicine. The internist assessed the plausibility of each identified complication and confirmed whether the intervention would likely have prevented it.

This validation process included a thorough examination of clinical notes, patient history, and any relevant diagnostic results. Once validated, these scenarios were used to model the potential economic benefits of the interventions, which involved calculating the costs associated with each avoided complication. The result of this validation is a total number of physician office visits, emergency room visits, and hospitalizations avoided due to the pharmaceutical interventions. For each of these medical services, an average cost was calculated based on the registered costs of these services in a private hospital in Costa Rica in 2023. The hospitalization event was associated with the average length of stay obtained from hospital records for estimation. Through this data, it was possible to calculate the total cost savings by reproducing other authors’ calculations.

The estimated economic value does not include medical fees, specialized treatments, laboratory tests, or hospital stays longer than three days. These aspects vary based on the specific patient’s condition and the physician assigned during their hospitalization.

Statistical analysis

Fisher’s exact test was employed on three separate tables in order to determine a possible relationship between the types of identified discrepancies and their respective classifications, the type of interventions carried out on healthcare professionals and their rate of acceptance, and finally the category of the identified DRPs and their potential severity. A descriptive analysis of the interviewed patient’s demographic characteristics was also performed. All statistical tests were done on RStudio version 4.3.0 (Posit Software, Boston, MA).

Ethics approval

Ethical approval to conduct this study was obtained from the Scientific Ethics Committee at Universidad de Costa Rica (reference number: CEC-242-2023).

## Results

Medication reconciliation upon admission

From the 457 patients interviewed during hospital admission for medication reconciliation, 302 (66.1%) reported chronic use of at least one medication before hospitalization. Of these, 53.0% of these had no prior medication reconciliation documented in their electronic health files for the current hospitalization. This group had a mean age of 66 years, with 51.0% of them being females. In total, 1,475 medications across several pharmacological categories were reviewed. Hypertension emerged as the predominant chronic condition, constituting 20.9% of the reconciled drug regimens. Notably, only 21.1% of the medications were recognized as high-risk, with oral hypoglycemic agents representing 32.8% of the total.

The discrepancies identified during the reconciliation phase are shown in Table 1. A total of 51 discrepancies were found, with medication omission being the most common at 60.8% and classified as indication DRPs. In most cases, these discrepancies were intentional, implying physicians were aware of the medications but opted not to include them in the inpatient regimen based on their clinical decision. Only 17.7% were unintentional discrepancies. A significant relationship was observed between the identified discrepancies and their relationship, as evidenced by Fisher's exact test.

**Table 1:** Discrepancies identified and classified in the medication reconciliation process at hospital admission

Discrepancies identified n (%)		Discrepancies classification n (%)			
		Indication	Safety	Effectiveness	Adherence
Omission of medication	31 (60.8)	30 (96.8)	-	1 (3.2)	-
Incorrect dose	4 (7.8)	-	2 (50.0)	2 (50.0)	-
Incorrect frequency	3 (5.9)	-	1 (33.3)	2 (66.7)	-
Inconsistencies in charts	13 (25.5)	13 (100.0)	-	-	-
Total	51 (100.0)	43 (84.3)	3 (5.9)	5 (9.8)	0 (0.0)
p-value	<0.000				

All identified discrepancies led to interventions by the clinical pharmacist, mainly directed towards the appropriate nursing, medical, or hospital pharmacy staff. The most frequent action involved relaying pertinent information to the clinical team, and, among these actions, 96.7% were directed at the nursing staff, mainly due to the omission of medication during their initial reconciliation process.

Interventions labeled as "partially accepted" were classified as such in the situations where the attending physician acknowledged that the noted discrepancies were deliberate and agreed to implement the intervention suggested, but only when they considered it appropriate based on the patient's condition. Interventions had a remarkable acceptance rate of 90.2%, as detailed in Table 2. A significant relationship was observed between the interventions performed on healthcare professionals and the percentage of interventions accepted by them, as evidenced by Fisher's exact test.

**Table 2:** Interventions made to health personnel based on the identified discrepancies and their acceptance during the medication reconciliation process *Adding a medication not used prior to admission

Interventions to healthcare professionals n (%)		Accepted interventions n (%)	
		Accepted	Partially accepted
Notification of relevant information	30 (58.8)	30 (100.0)	-
Dose modification	4 (7.8)	2 (50.0)	2 (50.0)
Frequency modification	3 (5.9)	-	3 (100.0)
Correction of inconsistency in charts	13 (25.5)	13 (100.0)	-
*Drug commission	1 (2.0)	1 (100.0)	-
Total	51 (100.0)	46 (90.2)	5 (9.8)
p-value	<0.000		

Hospital discharge medication analysis

For the 140 patients reviewed during hospital discharge, the average age was 63 years, 60.0% of whom were females. This demographic distribution mirrored the cohort during the admission reconciliation. A total of 572 medications underwent evaluation, with 18.9% classified as high-risk. Anticoagulants stood out as the most prevalent group.

A total of 56 DRPs were identified during discharge, implying that nearly 40% of the hospital discharges under revision were linked to a DRP. The categorization based on associated risk, drug category, and potential severity is outlined in Table 3. A significant relationship was observed between the characterization of the DRPs and their associated severity level, as evidenced by Fisher's exact test. Pharmaceutical interventions were initiated in response to each DRP, with drug interactions being the primary concern (Table 4).

**Table 3:** Categorization of the DRPs detected at discharge during the implementation of the Transitions of Care Medication Program DRPs: drug-related problems

Categorization of DRPs	Potential severity					Total
	Grade 1	Grade 2	Grade 3	Grade 4	Grade 5	n (%)
Safety	13	1	6	2	-	22 (39.3)
Effectiveness	7	3	-	-	3	13 (23.2)
Indication	4	1	6	-	-	11 (19.6)
Adherence	4	3	3	-	-	10 (17.9)
Total n (%)	28 (50)	8 (14.3)	15 (26.8)	2 (3.6)	3 (5.4)	56 (100.0)
p-value	0.0143					

**Table 4:** Interventions made to patients based on the discrepancies identified during the discharge process and its percentage of acceptance

Intervention to patients and/or support network n (%)		Accepted interventions n (%)
Education (medication)	189 (70.5)	189 (100)
Education (disease)	3 (1.1)	3 (100)
Medication identification	56 (20.9)	56 (100)
Medication schedule	20 (7.5)	20 (100)
Total	268 (100.0)	268 (100)

Furthermore, 268 pharmaceutical interventions were directly extended to patients and their support networks; in some cases, a single patient might have required more than one of these interventions (Table 4).

Regarding patient education of their medication, particular emphasis was placed on ensuring the accurate administration and proper dosage of prescribed drugs, as well as potential adverse effects and their appropriate management. Similarly, when it came to educating patients about their medical conditions, the focus was on providing recommendations for non-pharmacological approaches to managing specific diseases.

As shown in Table 5, out of a total of 56 interventions performed on healthcare professionals, 78.6% were accepted, which demonstrates the relevance of clinical pharmacotherapeutic monitoring in the patient transition process.

**Table 5:** Interventions made to health personnel based on the discrepancies identified during the discharge process and its percentage of acceptance

Intervention to healthcare professionals n (%)		Accepted interventions n (%)
Report of drug-drug interaction	13 (23.2)	9 (69.2)
Adherence strategies	11 (19.6)	11 (100.0)
Report of therapeutic duplicity	7 (12.5)	4 (57.1)
Dosage modification	4 (7.1)	4 (100.0)
Education	3 (5.3)	3 (100.0)
Correction of inconsistency in charts	3 (5.3)	3 (100.0)
Report of medication error	3 (5.3)	3 (100.0)
Drug suspension	3 (5.3)	1 (33.3)
Drug substitution	3 (5.3)	1 (33.3)
Treatment duration modification	2 (3.6)	2 (100.0)
Drug commission	2 (3.6)	2 (100.0)
Report / management of drug allergies	2 (3.6)	2 (100.0)
Total	56 (100.0)	44 (78.6)

Economic implications of interventions

The analysis revealed that 50% of the DRPs could have led to emergency service admissions, resulting in a total cost savings of approximately $4,995; 31.8% might have led to outpatient visits to specialist physicians, representing an estimated total savings of $1,680; and the remaining 18.2% could have needed hospital admissions, leading to approximate savings of $14,335. Thus, the total estimated amount saved from these interventions amounts to $21,010 (Table 6).

**Table 6:** Cost of potential medical services avoided through accepted pharmaceutical interventions *Represents an average daily value of hospital stay. ·Does not consider medical fees of other expenses (18.000 USD dollars per patient’s hospital stay), so the amount may vary extensively.

Potential avoided medical service n (%)		Cost per unit	Total cost	
Emergency care	22 (50)	$227.04		$4.994,88
Hospital admission	8 (18.2)	$597.27^*·^	$14.334,48	
Specialist outpatient consultation	14 (31.8)	$120.00	$1.680,10	
Total	44 (100)		$21.009,50	

## Discussion

A significant finding from our study was that 66.1% of patients reported chronic use of at least one medication before hospitalization. This reflects the global trend of increasing chronic diseases, resulting in greater long-term medication use and polypharmacy [24]. Our approach guarantees that the monitoring and effective administration of chronic therapy is tailored to each patient's hospitalization context. Furthermore, it helps prioritize their discharge plan and consider any high-risk medications [12,25,26]. If discrepancies arise regarding such medicines, it is crucial to promptly address the issue within a defined timeframe to minimize the potential risk of harm [27].

The absence of prior medication reconciliation in the electronic health records for 53.0% of these patients not only highlights the necessity for improvement in the hospital’s medication reconciliation process but also underscores the importance of involving pharmaceutical personnel (including clinical pharmacists, hospital staff, interns, and technicians) in this endeavor. Collaborative efforts from pharmacy staff can streamline and enhance this practice, serving as a bridge for effective communication among the various clinical team members responsible for patient care [28].

As reported in this study, 268 pharmaceutical interventions were directly provided to patients and their support networks at the time of discharge, in addition to 56 interventions directed toward the clinical team. Moreover, the prevalence of DRPs identified during this stage, which approached 40%, underscores the complexity of medication management during TOC and highlights the critical role of pharmacists in ensuring patient safety. [26,29-31]. Interventions made, not only rectify the medication discrepancies but also contribute to improving patient outcomes and increasing patient satisfaction [25,32].

The high acceptance rate of pharmaceutical interventions during the reconciliation process (90.2%) and discharge stage (78.6%) highlights the collaborative nature of healthcare and the trust placed in pharmacists. While this study did not ascertain the impact of the pharmacist-led TOC protocol on readmissions, given the methodology employed, it does demonstrate its favorable influence on patient safety.

The TOC interventions, as seen in the literature, can lead to economic benefits related to the patient and also for the healthcare systems [33]. This study determined a total savings of $21,009.50 based on the interventions made by clinical pharmacists solely during the discharge process. It is important to highlight that the economic appraisal linked to hospital admission, viewed as a potential foregone medical service, is based on the average basic cost of hospitalization for three days.

Pharmacists can play a pivotal role in promoting medication adherence and effective medication reconciliation. However, the literature has yet to pinpoint a single pharmacist intervention as the most effective in enhancing TOC, which shows the need for further research to determine best practices in pharmacist-led TOC programs [34-36]. In this specific domain, this study unveils the scene for further investigation into medication care transitions, presenting an avenue for a more systematic examination of the clinical and economic ramifications inherent in the sequence of "hospital admission-hospitalization-hospital discharge-post-discharge follow-up." Particularly in countries like Costa Rica, where research on medication management transitions remains relatively underexplored, this work paves the way for deeper insights into this critical area at a local level, where clinical indicators, including hospital readmissions, could potentially be determined and studied.

Limitations

The study encountered significant limitations in conducting simultaneous data collection for medication reconciliation and discharge processes due to staffing constraints. With only one clinical pharmacist available, it was not possible to collect data for both processes during the same timeline. Additionally, post-discharge follow-up could not be conducted due to the same personnel limitations, impeding the assessment of long-term medication program effectiveness. These constraints underscore the importance of adequate staffing and resources for thorough data collection and follow-up in medication programs.

The calculation of costs does not include additional medical fees or expenses, such as charges for medical consultations, medications, surgical procedures, and any other related costs. This limitation results from the variability in expenses during hospital stays, shaped by the patient's circumstances and health condition. Consequently, attempting to establish an averaged value while factoring in these variables proves to be impractical.

## Conclusions

In navigating global healthcare challenges, pharmacist-led TOC programs play a vital role in ensuring secure medication management during transitions of care. These programs offer substantial clinical benefits by preventing and rectifying medication discrepancies and safeguarding patient safety. Additionally, their implementation shows promise in generating positive economic outcomes. Future research can delve into the long-term impacts of pharmacist-led interventions and optimize TOC medication programs further.

## Appendices

Appendix A

**Table 7:** Checklist of procedures for reviewing and analyzing patient pharmacotherapy during hospital admission and discharge

Aspect under revision	Check
Therapeutic indication	
Confirm the diagnosed condition or indication for each medication.	
Evaluate whether the prescribed medication aligns with evidence-based guidelines for the condition.	
Dosage	
Verify the prescribed dosage of each medication.	
Cross-check with recommended dosages based on patient characteristics (age, weight, renal/hepatic function).	
Identify higher or lower dosages being administered.	
Frequency of administration	
Verify the prescribed dosage of each medication.	
Assess the appropriateness of the dosing schedule based on pharmacokinetics, therapeutic goals, and patient preference.	
Drug interactions	
Identify potential interactions between prescribed medications.	
Utilize drug interaction databases or reference sources to assess the significance and management of interactions.	
Determine the clinical significance of potential drug interactions.	
Adverse effects	
Investigate potential adverse effects associated with each medication.	
Consider patient-specific factors that may increase susceptibility to adverse reactions (e.g., allergies, comorbidities).	
Determine the clinical significance of potential adverse effects.	
